# Fistule vesico cutanée post traumatique

**DOI:** 10.11604/pamj.2017.28.147.12131

**Published:** 2017-10-16

**Authors:** Othmane Yddoussalah, Harrison Sumba

**Affiliations:** 1 Service d'Urologie B, CHU Ibn Sina, Faculté de Médecine et de Pharmacie de Rabat, Université Mohamed V, Maroc

**Keywords:** Fistula, vesicocutaneous, post traumatic

## Abstract

We here report the case of a 43-year old motorcyclist who was initially examined in the emergency department for injuries following a road accident with a car. The patient had pelvic trauma with extensive symphyseal disjunction (A). He underwent orthopedic treatment based on weight compensation. During the two weeks immediately following the trauma, patient's evolution was marked by the occurrence of fistulous orifice at the level of the inner right thigh (B) associated with urinary outflow and wounds due to local skin maceration around the fistula. Radiologically, abdominopelvic CT scan at extended injection-to-scan acquisition time showed contrast extravasation laterally, into the bladder and at the level of the right thigh root due to subperitoneal rupture of the bladder (C). Uretrocystography objectified symphyseal disjunction visible on the non-contrast abdominal X-ray film. Moreover, uretrocystography showed fistulous track after uterovesical opacification (D). Standard laboratory tests were normal. Given the subperitoneal rupture of the bladder and the absence of associated lesions, conservative treatment by prolonged bladder drainage with urethral catheter and local care of the fistulous orifice were performed. Patient's evolution was marked by complete drying up of the fistula.

## Image en médecine

Un homme âgé de 43 ans était vu en urgence initialement pour prise en charge d'accident de la voie publique motocycliste heurte par une voiture. Responsable d un traumatisme du bassin avec une disjonction symphysaire importante (A). Traitée orthopédiquement par une décharge. L évolution a été marque par l apparition d un orifice fistuleux localise au niveau de la face interne de la cuisse droite (B) dans les deux semaines suivant le traumatisme, avec écoulement urinaire et des stigmate de macération locale autour de la fistule. Sur le plan radiologique, un TDM abdominopelvien avec temps tardive avait montre une extravasation du produit de contraste en latero vésicale et au niveau de la racine de la cuisse droite en rapport avec une brèche vésicale sous péritonéale (C). Une urétrocystographie réalisée a objective une disjonction symphysaire manifeste sur le cliche sans préparation. Avec la présence d un trajet fistuleux après opacification uretro vésicale (D). Les examens biologiques standard étaient normaux. Devant le caractère sous péritonéale de la brèche vésicale et l absence de lésion associes, on a opte pour un traitement conservateur a savoir un drainage vésical prolongé par une sonde urétrale. Avec des soin locaux au niveau de l orifice fistuleux. L évolution a été marque par l obtention de l'assèchement définitif de la fistule.

**Figure 1:**
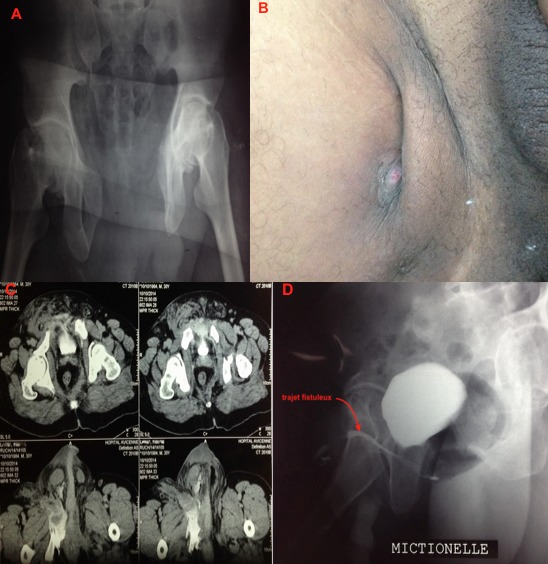
(A) radiographie du bassin montrant une importante disjonction symphysaire; (B) orifice fistuleux sur la face interne de la cuisse droite; (C) coupes tomodensitométriques pelviennes au temps tardif avec extravasation du produit de contraste à travers une plaie vésicale; (D) urétrocystographie au temps mictionnel objectivant un trajet fistuleux vesicocutane

